# An investigation of transportation practices in an Ontario swine system using descriptive network analysis

**DOI:** 10.1371/journal.pone.0226813

**Published:** 2020-01-10

**Authors:** Dylan John Melmer, Terri L. O’Sullivan, Amy L. Greer, Zvonimir Poljak

**Affiliations:** Department of Population Medicine, University of Guelph, Guelph, ON, Canada; Tsinghua University, CHINA

## Abstract

The objectives of this research were to describe the contact structure of transportation vehicles and swine facilities in an Ontario swine production system, and to assess their potential contribution to possible disease transmission over different time periods. A years’ worth of data (2015) was obtained from a large swine production and data management company located in Ontario, Canada. There was a total of 155 different transportation vehicles, and 220 different farms within the study population. Two-mode networks were constructed for 1-,3-, and 7-day time periods over the entire year and were analyzed. Trends in the size of the maximum weak component and outgoing contact chain over discrete time periods were investigated using linear regression. Additionally, the number of different types of facilities with betweenness >0 and in/out degree>0 were analyzed using Poisson regression. Maximum weekly outgoing contact chain (MOCC_w_) contained between 2.1% and 7.1% of the study population. This suggests a potential maximum of disease spread within this population if the disease was detected within one week. Frequency of node types within MOCC_w_ showed considerable variability; although nursery sites were relatively most frequent. The regression analysis of several node and network level statistics indicated a potential peak time of connectivity during the summer months and warrants further confirmation and investigation. The inclusion of transportation vehicles contributed to the linear increase in the maximum weekly weak component (MWC_w_) size over time. This finding in combination with constant population dynamics, may have been driven by the differential utilization of trucks over time. Despite known limitations of maximum weak components as an estimator of possible outbreaks, this finding suggests that transportation vehicles should be included, when possible and relevant, in the evaluation of contacts between farms.

## Introduction

Network analysis as a tool for epidemiologists has been around at least since the 1980s [1]. It has also become a frequently utilized approach within veterinary epidemiology to help expand our understanding of disease transmission for production animal research [2–8]. Knowledge about the movement of animals is essential for understanding the potential for diseases to spread [9]. In swine populations, this movement is particularly frequent in areas where strict age segregation and external sources of replacement animals are common practices. Transportation vehicles involved in animal movement can also provide a means for disease spread between farms [10,11]. As an example, a study published in 2014 found that during the 2013 Porcine Epidemic Diarrhea (PED) outbreak in the United States, transportation vehicles were an important fomite in the spread of the PED virus (PEDV) [12]. In previous work, it has also been found that sharing transport trucks could increase the number of infected farms during a simulated epidemic by 44% [13]. This was compared to simulated epidemics that did not consider transportation vehicles [13]. For such reasons, transportation practices are often being considered an important aspect of overall biosecurity on swine establishments. One aspect of transportation biosecurity is focused on proper cleaning and disinfection practices for individual vehicles, so that the risk of contamination is reduced [14]. While it would be ideal to have all transportation vehicles adequately sanitized before each new load, this practice can be limited due to substantial cost or lack of time. Directly related to the biosecurity of individual vehicles is the actual usage of transportation vehicles. This can include the planning of trips between different premises and premise types. This aspect of biosecurity is less studied in the current literature. Previous work has investigated the role of just animal movements within the same system, yet the incorporation of transportation vehicles was ignored [4]. Therefore, the addition of these potential fomites may provide further insight into disease transmission potential within production systems over various time periods. [12]. Additional research has also utilized network measures to understand the potential for disease transmission, and how utilizing these measures can aid in the mitigation of disease transmission [4,7]. Thus, one of the objectives of this study was to describe the contact structure of transportation vehicles and swine facilities in a study population which consisted of multiple swine facilities within an Ontario swine production system. Furthermore, we aimed to understand the effect of swine movements on disease transmission potential over discrete 1-,3-, and 7-day time periods. This objective has been accomplished by: (i) generating a series of networks that were based on distinct time periods of fixed length over the entire year, (ii) determining selected node- and network-level characteristics over these distinct fixed time periods, and (iii) summarizing the characteristics of these networks descriptively or analytically over the entire study period.

## Materials & methods

### Study population

Via connections built over the years with the Ontario swine industry, daily animal movement data were obtained from a swine production and data management company located in Ontario (Canada); which included all relevant data from January 1^st^ 2015 to December 31^st^ 2015. Animal movements were confined to southwestern Ontario, with some movements to locations in other provinces and exports to other countries in North America. The dataset contained unique identification of several types of swine establishments, along with license plates of transportation vehicles involved in animal movement among these establishments. For confidentiality, the locations and identifiers have been kept anonymous along with the data provider. To inquire about the data utilized please contact the Research Ethics Board of the University of Guelph at reb@uoguelph.ca.

The raw data is the same data utilized in previous work [4], which consisted of 5398 unique movements (edges), with 16 descriptive variables defining edge attributes. The nodes or facilities considered were stratified into: sow herds, nurseries, finisher facilities, and abattoir sites, and included the addition of individual, unique transportation vehicles.

The data also contained records of pig movements from or to business entities that could potentially represent more than one location under the same unique identifier. However, details about specific locations were not part of the dataset. Such nodes were classified into three distinct groups for the purposes of this analysis: Company Internal (CI), Company External (CE), and Company Export (CE_X_). These three types of nodes were defined previously [4]. In brief, Company Internal described nodes located in Canada which were, over the period of the entire year, only a destination for movements from facilities within the production system. Company Export was used to describe an identical type of destination for animal movement as CI, except that the destination was outside of Canada. Company External was used to describe entities that, over a period of the entire year, served as source of animals for nodes within the production system, but could also be destination for animals from nodes within the production system [4]. This resulted in the network that consisted of 375 unique facilities, in which truck license plates were added to the swine production related sites.

### Data management

In order to construct the new truck dataset from the original data, some observations were excluded due to a lack of information on truck license plates or transportation companies used. Specifically, the missing data involved movements from 3 sow herds, and a single facility that was classified as CE. The total number of observations removed from the modified data set was 137 movements or ~4% of the observations before the data set was converted into the modified data utilized for analysis. Data management and analysis was carried out with R x64, version 3.3. [15], using packages *doBy* [16], and *lubridate* [17].

Two-mode daily, three-day, and weekly directed networks were then constructed from movement data using functionality available in the package *igraph* [18]; wherein swine facilities represented the first mode and transportation vehicles represented the second mode. These time frames were utilized to account for possible transportation vehicle sanitation practices. In addition, all days within the year were accounted for within the analysis, which included days in which no swine movements occurred and therefore no networks could be constructed over the period of interest. Following this, node characteristics were examined descriptively and analytically over time.

### Descriptive network analysis: Daily and three-day two-mode networks

Directed two-mode daily and 3 day-networks were generated and evaluated for the entire year. For both time scales, the node centrality measures of interest were: in- and out-degree, betweenness, and the number of movements (daily). The number of daily movements is a measure of the number of shipments occurring per facility type. However, the number of observed movements for trucks was halved, as a truck is only used once per shipment between the two swine establishments. This single movement was recorded twice in the dataset (i.e., truck first served as a recipient and then as a source of movement for a single shipment between the two swine establishments).

For both time scales, the network-level measures of interest were the size of maximum weak component (WC) and maximum strong component (SC) in a given time interval. Finally, one-mode daily networks of contacts among swine establishments were constructed from the two-mode daily networks in order to better understand the daily contact between establishments that occur through transportation vehicles involved in animal movement.

### Descriptive network analysis: Weekly networks

Weekly network level measures of interest were strong and weak component size and largest outgoing contact chain (OCC_w_). Contact tracing within the OCC measures were determined through the package *EpiContactTrace* [19]. Additionally, node-centrality measures of interest were: in- and out-degree, and betweenness. Following the calculation of weekly node- and network-level measures; select measures were further aggregated to a weekly level to describe any trends over time. Specifically, the size of the largest weak component and OCC_w_, demographic characteristics of the nodes involved in the largest weak components and OCC_w_, number of trucks, nursery and finisher sites with the betweenness >0, and number of trucks, finisher and nursery sites where both in-degree and out-degree were >0 in each week. For the current analysis, it was also of interest to examine which types of premises individual trucks typically connect on a weekly basis. Thus, such pairs of facilities were determined, and their demographics was summarized.

The magnitude of the weekly maximum weak component size over time was analyzed using linear regression. For nursery, finisher and truck facility types separately, the number of nodes with betweenness values >0 over time, and number of nodes with in- and out-degree > 0 over time was evaluated using Poisson regression, with week number as an explanatory variable. In all analyses, linear and quadratic effect of week numbers were evaluated as explanatory variables. Week 53 data was dropped from all analysis a priori, with the rationale that it was an incomplete week. All statistical analysis was conducted through *stats* version 3.3.2 [15] and model fit, normality and homoscedasticity were examined where applicable. In particular to the Poisson regression models, the deviance goodness-of-fit was calculated via the core R stats package utilizing the *pchisq* function [15].

## Results

### Study population

The facility demographics for the two-mode network (n = 375) using license plates as one of the modes consisted of 155 (~41%) trucks or unique license plates belonging to 13 transportation companies, 30 sow herds (~8%), 46 nursery facilities (~12%), 119 finisher facilities (~32%), and 10 abattoir sites (~3%). Additionally, 2 nodes were classified as CI, 9 nodes as CE and 4 nodes as CE_X_ which all together accounted for ~4% of the facilities.

### Daily and three-day two mode networks: Node centrality measures and movements

Descriptive statistics of a degree (d), in-degree (d_i_), and out-degree (d_o_) calculated on a daily, and 3-day basis are displayed in Table 1. On a daily basis, abattoirs had the highest average in-degree (Table 1). However, trucks had the largest maximum in-degree (d_i_ = 12) followed by abattoirs (d_i_ = 9) and finisher facilities (d_i_ = 3). Over the entire year, trucks ranged from a maximum daily in-degree of 1 to 12 with a median maximum value of 3. Furthermore, trucks shared the highest average out-degree status with nursery, finisher and sow facilities (Table 1). Conversely, finisher facilities had the highest maximum daily out-degree value (d_o_ = 6), followed by trucks (d_o_ = 4) by and nursery facilities (d_o_ = 3). In addition, the maximum daily out-degree status ranged from 1 to 4 with a median of 3. Similar patterns have been observed for the 3-day networks.

**Table 1:** Descriptive statistics of the node-level centrality measures calculated from daily, 3-day and weekly networks during 2015 in an Ontario swine production system, stratified by the type of facility.

		*Mean*			*Standard deviation*			*Maximum*		
*Time Frame*	Facility type	Betweenness	In-Degree	Out-Degree	Betweenness	In-degree	Out-Degree	Betweenness	In-degree	Out-Degree
Day*	**Abattoir**	0.0E+00	6.5E-01	0.0E+00	0.0E+00	1.4E+00	0.0E+00	0.0E+00	9.0E+00	0.0E+00
	**Company Export**	0.0E+00	8.5E-03	0.0E+00	0.0E+00	1.0E-01	0.0E+00	0.0E+00	2.0E+00	0.0E+00
	**Company External**	0.0E+00	5.9E-03	1.8E-02	0.0E+00	7.6E-02	1.4E-01	0.0E+00	1.0E+00	2.0E+00
	**Company Internal**	0.0E+00	6.2E-03	0.0E+00	0.0E+00	7.9E-02	0.0E+00	0.0E+00	1.0E+00	0.0E+00
	**Finisher**	1.1E-02	1.8E-02	8.4E-02	3.0E-01	1.4E-01	3.2E-01	2.3E+01	3.0E+00	6.0E+00
	**Nursery**	2.6E-02	5.5E-02	5.8E-02	4.4E-01	2.3E-01	2.5E-01	2.0E+01	2.0E+00	3.0E+00
	**Sow**	7.2E-04	7.5E-03	8.5E-02	5.1E-02	8.6E-02	2.8E-01	4.0E+00	1.0E+00	2.0E+00
	**Truck**	1.3E-01	9.9E-02	7.4E-02	7.4E-01	4.3E-01	3.0E-01	2.4E+01	1.2E+01	4.0E+00
***Three-day***										
	**Abattoir**	0.0	1.5	0.0	0.0	2.7	0.0	0.0	13.0	0.0
	**Company Export**	0.0	0.0	0.0	0.0	0.2	0.0	0.0	2.0	0.0
	**Company External**	0.0	0.0	0.0	0.0	0.1	0.2	0.0	1.0	2.0
	**Company Internal**	0.0	0.0	0.0	0.0	0.1	0.0	0.0	1.0	0.0
	**Finisher**	0.2	0.0	0.2	2.3	0.2	0.6	70.5	3.0	6.0
	**Nursery**	0.5	0.1	0.1	4.0	0.4	0.4	109.0	3.0	3.0
	**Sow**	0.0	0.0	0.2	0.2	0.1	0.4	8.0	1.0	2.0
	**Truck**	0.7	0.3	0.2	4.7	0.8	0.5	138.0	12.0	8.0
***Week***										
	**Abattoir**	0.0	5.4	0.0	0.0	5.1	0.0	0.0	21.0	0.0
	**Company Export**	0.0	1.2	0.0	0.0	0.4	0.0	0.0	2.0	0.0
	**Company External**	0.0	0.2	0.8	0.0	0.4	0.5	0.0	1.0	2.0
	**Company Internal**	0.0	1.0	0.0	0.0	0.0	0.0	0.0	1.0	0.0
	**Finisher**	1.5	0.3	1.6	10.1	0.6	1.2	214.9	3.0	8.0
	**Nursery**	4.4	0.7	0.8	22.3	0.7	0.8	353.0	3.0	4.0
	**Sow**	0.0	0.1	1.1	0.6	0.3	0.5	24.0	1.0	2.0
	**Truck**	4.2	2.7	1.8	24.5	2.0	1.4	752.0	12	10.0

*Numbers provided in the science notation to provide more details

Average betweenness values for the daily and three-day interval networks are displayed in Table 1 as well. Excluding the weekly networks, where nursery facilities had the highest average betweenness, trucks had the highest betweenness values, followed by nursery facilities, and then by finisher sites on a daily and three-day level (Table 1). Trucks had the highest maximum betweenness values for all three time periods evaluated (Table 1).

### Daily and three-day two mode networks: Network measures

Maximum daily weak components (MWC_D_) ranged from 3 nodes to 46 nodes. The demographics of the maximum daily weak component are displayed in Tables 2 and 3 as a proportional percent occurrence, and as a percent occurrence based on the total number of nodes of each facility type present in the study population over the entire year, respectively. Daily strong components (SC_D_) ranged from a minimum of 2 nodes across multiple days, to a maximum of 3 nodes in days 170 and 180.

**Table 2:** Proportional representation of each facility type in the maximum weak component obtained from daily and 3-day 2-mode networks over the entire 2015, expressed as descriptive statistics of the percent of each facility type in the maximum weak component.

	*Daily networks*				*Three-day networks*			
*Facility type*	mean	min	max	sd	mean	min	max	sd
***Finisher***	47.0	0.0	64.0	11.0	40.0	0.0	64.0	16.0
***Truck***	35.0	14.0	60.0	6.0	29.0	0.0	45.0	11.0
***Abattoir***	12.0	0.0	33.0	6.0	7.0	0.0	29.0	4.0
***Nursery***	3.0	0.0	50.0	8.0	7.0	0.0	33.0	7.0
***Sow***	2.0	0.0	50.0	7.0	5.0	0.0	50.0	7.0
***Company External***	0.0	0.0	8.0	1.0	0.0	0.0	3.0	1.0
***Company Internal***	0.0	0.0	4.0	0.0	0.0	0.0	3.0	0.0
***Company Export***	0.0	0.0	3.0	0.0	0.0	0.0	6.0	1.0

**Table 3:** Proportional representation of each facility type in the maximum weak component obtained from daily and 3-day 2-mode networks over the entire 2015, expressed as a descriptive statistic of the percent of each facility type in the study population.

	Daily networks				Three-day networks			
Facility type	mean	min	max	sd	mean	min	max	sd
**Finisher**	6.0	0.0	16.0	3.0	16.0	0.0	28.0	7.0
**Truck**	3.0	1.0	10.0	2.0	10.0	1.0	20.0	4.0
**Abattoir**	18.0	0.0	50.0	10.0	35.0	0.0	70.0	15.0
**Nursery**	1.0	0.0	17.0	3.0	11.0	0.0	37.0	11.0
**Sow**	1.0	0.0	13.0	3.0	12.0	0.0	43.0	13.0
**Company External**	1.0	0.0	11.0	3.0	3.0	0.0	22.0	6.0
**Company Internal**	0.0	0.0	50.0	3.0	1.0	0.0	50.0	6.0
**Company Export**	0.0	0.0	25.0	1.0	0.0	0.0	25.0	3.0

Three-day maximum weak components (MWC_TD_) ranged from 6 to 92 nodes in size, while the three-day strong component (SC_TD_) ranged from 2 to 3 nodes in size. The demographics of the MWC_TD_ can be seen in Tables 2 and 3, respectively. The percentages of each facility type with the MWC_TD_ are calculated using the respective number of each facility type in the weak components as the numerator. However, the denominator changes between Tables 2 and 3, specifically, Table 2 utilizes the total number of facilities within the MWC_TD_ while Table 3 percentages are calculated based on the total number of each facility type within the study population.

### Weekly networks: Node centrality

Weekly in and out degree values for the truck network can be seen in Table 1. On a weekly basis, trucks had the highest average out degree values, and the second highest average in degree values (Table 1). Additionally, average betweenness values for trucks were the second largest in magnitude next to nursery facilities (Table 1).

The number of trucks, finisher, and nursery facilities with betweenness values > 0 and both, in and out degree values >0 in a given week were tested for association with time. Number of trucks with high betweenness and in/out-degree showed a significant linear relationship with time (deviance chi-square GOF *P =* 0.9) (Table 4). In addition, the number of finisher and nursery facilities showed a significant quadratic relationship with week for the number of facilities with a betweenness>0, along with the number of facilities with an in/out degree value >0 (deviance chi-square GOF *P =* 0.9) (Table 4).

**Table 4:** Results of Poisson regression using the weekly count of three different types of facilities with betweenness or in/out degree exceeding 0 as an outcome, and different functional forms of time as explanatory variables.

Variable	Facility Type	Variable	Coefficient	Standard error	p
**Betweenness Count**	Finisher	Intercept	1.0377		
		Week	4.375e-02	0.0185	0.02
		Week^2	-6.83e-04	0.0003	0.04
	Nursery	Intercept	3.757e-01		
		Week	6.365e-02	0.0232	0.02
		Week^2	-9.52e-04	0.0004	<0.01
	Truck	Intercept	3.3124		
		Week	5.89e-03	0.0016	<0.01
**In-Out Degree Count**	Finisher	Intercept	1.0579		
		Week	4.642e-02	0.0182	0.01
		Week^2	-7.52e-04	0.0003	0.02
	Nursery	Intercept	3.412e-01		
		Week	6.806e-02	0.0209	<0.01
		Week^2	-1.01e-03	0.0004	0.01
	Truck	Intercept	3.3113		
		Week	6.03e-03	0.0016	<0.01

### Weekly networks: Network measures and contacts/movements

Maximum weekly strong components (MSC_w_) had a maximum of seven nodes at week 39, and a minimum of two nodes across 25 different weeks. The maximum weak component (MWC_w_) on a weekly basis for the networks including transportation vehicles ranged from 3 nodes in multiple weeks, to 123 nodes in week 43. The average size of the MWC_w_ across the entire year was 100.9 or ~101 (26.93%) nodes (sd = 12.2). The size of the MWC_w_ also had a significant linear relationship with week, (β = 0.34, p*<*0.001; Table 5). There was also a significant linear increase in the number of trucks being used throughout the year on a weekly basis, (β = 0.19, p*<*0.001; Table 5). Contrary to truck utilization, all other facility types utilized per week within the network did not demonstrate any significant changes (p<0.05) when evaluated using linear regression models. No significant quadratic association of time could be detected for the latter two outcomes (p>0.10).

**Table 5:** Linear regression results for weekly maximum weak component, outgoing contact chain, and truck utilization per week, different functional forms of explanatory variables displayed were relevant.

Variable	Variable	Coefficient	Standard error	p
	Intercept	91.4072	2.5937	<0.01
**Maximum Weak Component**	Week	3.475e-01	0.0851	<0.01
**Maximum Outgoing Contact Chain**	Intercept	10.5496	1.782	<0.01
	Week	4.444e-01	0.0155	<0.01
	Week^2	-7.5e-03	0.002	0.01
**Trucks Utilized Per Week**	Intercept	27.15158	0.9938	<0.01
	Week	0.19457	0.0326	<0.01

**Fig 1:**
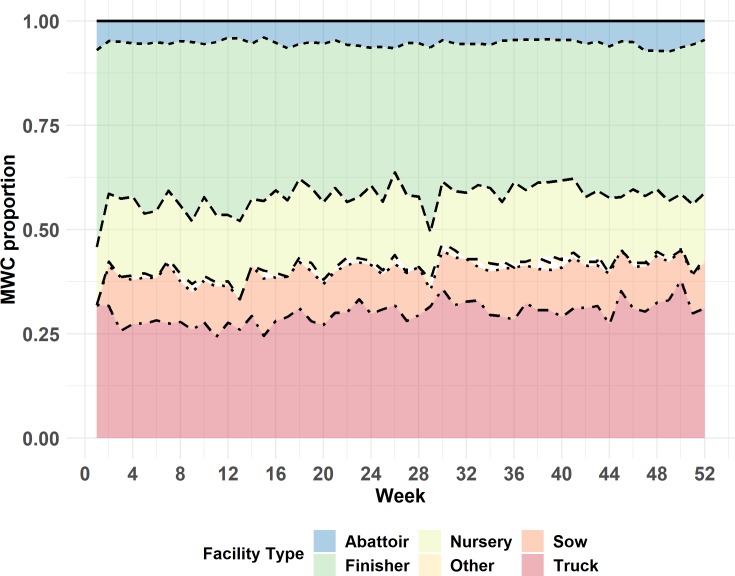
Proportional representation of each facility type within weekly maximum weak components for the year of 2015, calculated on the basis of two-mode network. The total number of nodes in a specific week in the MWC has been used as a denominator.

**Fig 2:**
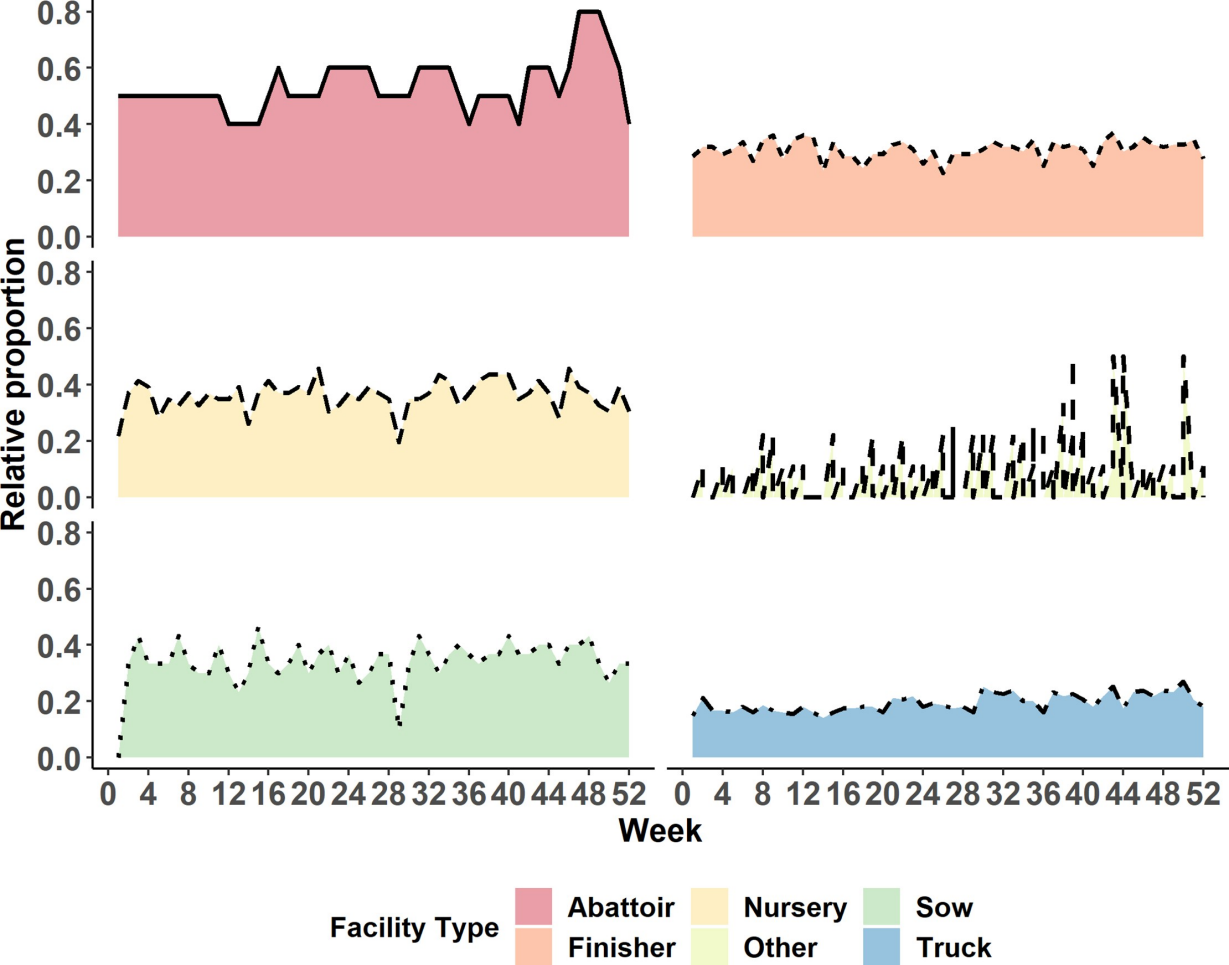
Relative occurrence of specific facility types in maximum weekly weak components over the entire year of 2015, when expressed as a proportion of each facility type in the study population.

The demographics of the MWC_w_ can be seen in Figs 1 and 2. Fig 1 displays the proportional representation of each facility type within the maximum weekly weak component over the entire year of 2015. Therefore, this proportion should always sum to one. Fig 2 displays the cumulative proportion of each facility type within the maximum weekly component, when expressed as proportion of each facility type in the study population.

**Fig 3:**
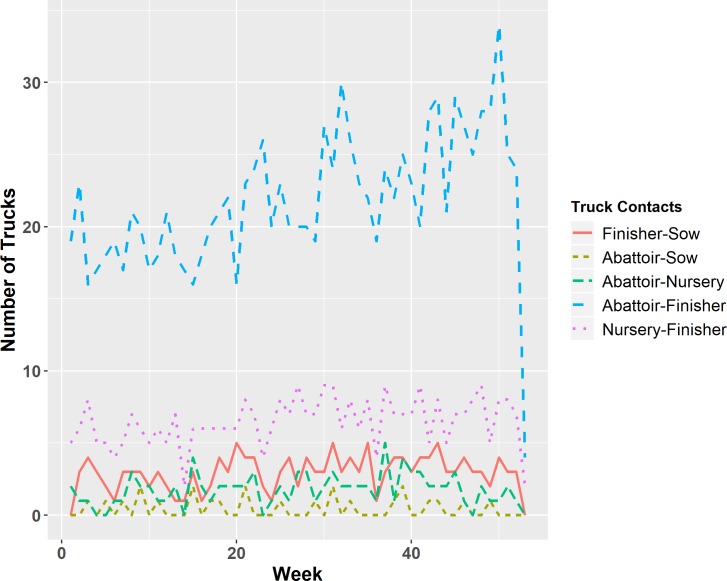
The total number of trucks that had contact with a specific combination of facility types within a given week.

Specific truck connections were also investigated across subsequent weeks. Fig 3 displays the number of trucks that had contact with specific combination of facility types within a given week. Notably, more trucks were involved in finisher-abattoir, and nursery-finisher type of movement than any other contact type. However, trucks in this network could occasionally be involved in a potentially riskier practice such as contact involving finisher and sow facilities, and an abattoir and sow facility within a given day and week (Fig 3). Similar results were seen on a daily level as well (S1 Fig)

**Fig 4:**
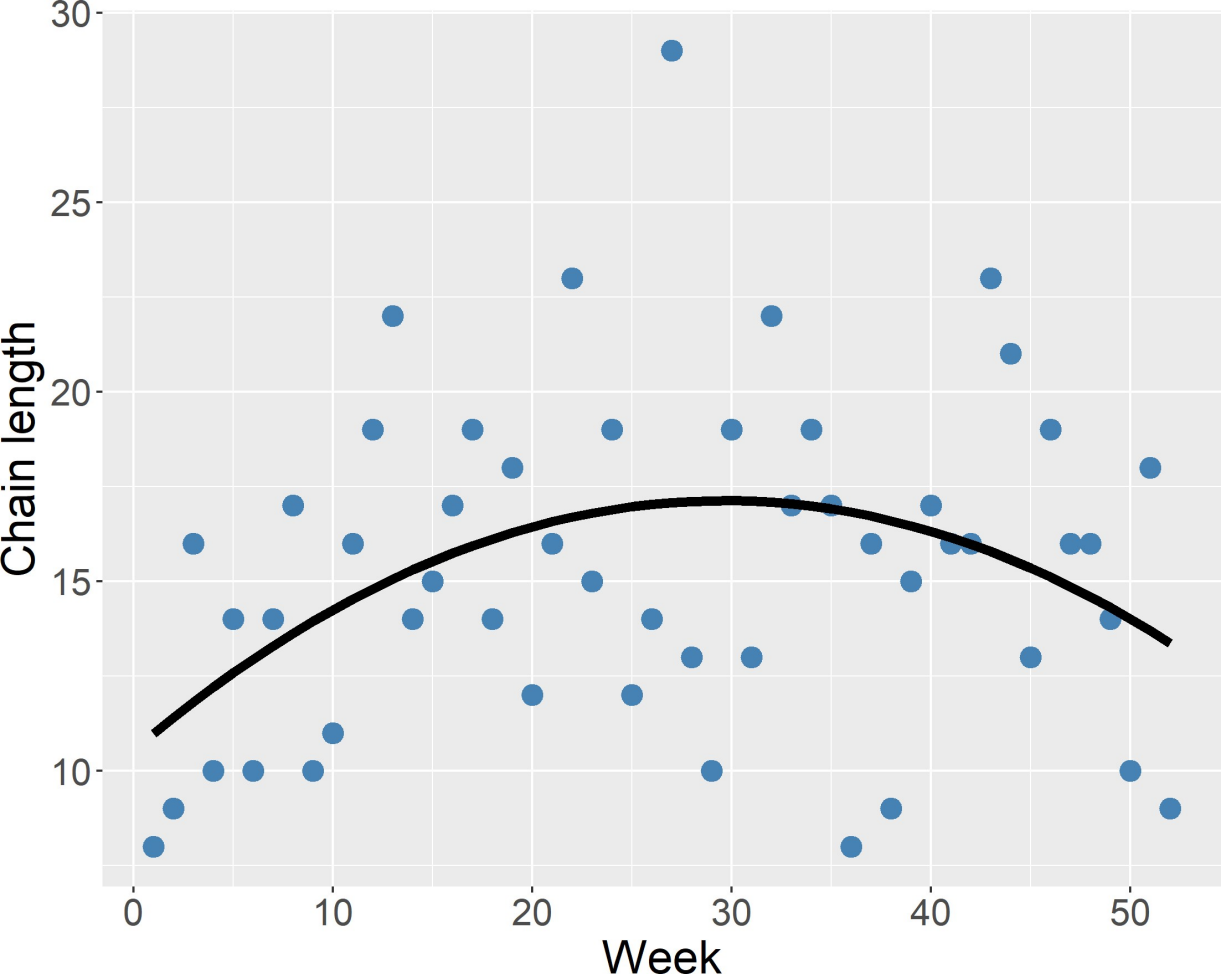
Weekly maximum outgoing contact chain length by week of the year. Chain length displayed a significant quadratic association with week, *P =* 0.01.

**Fig 5:**
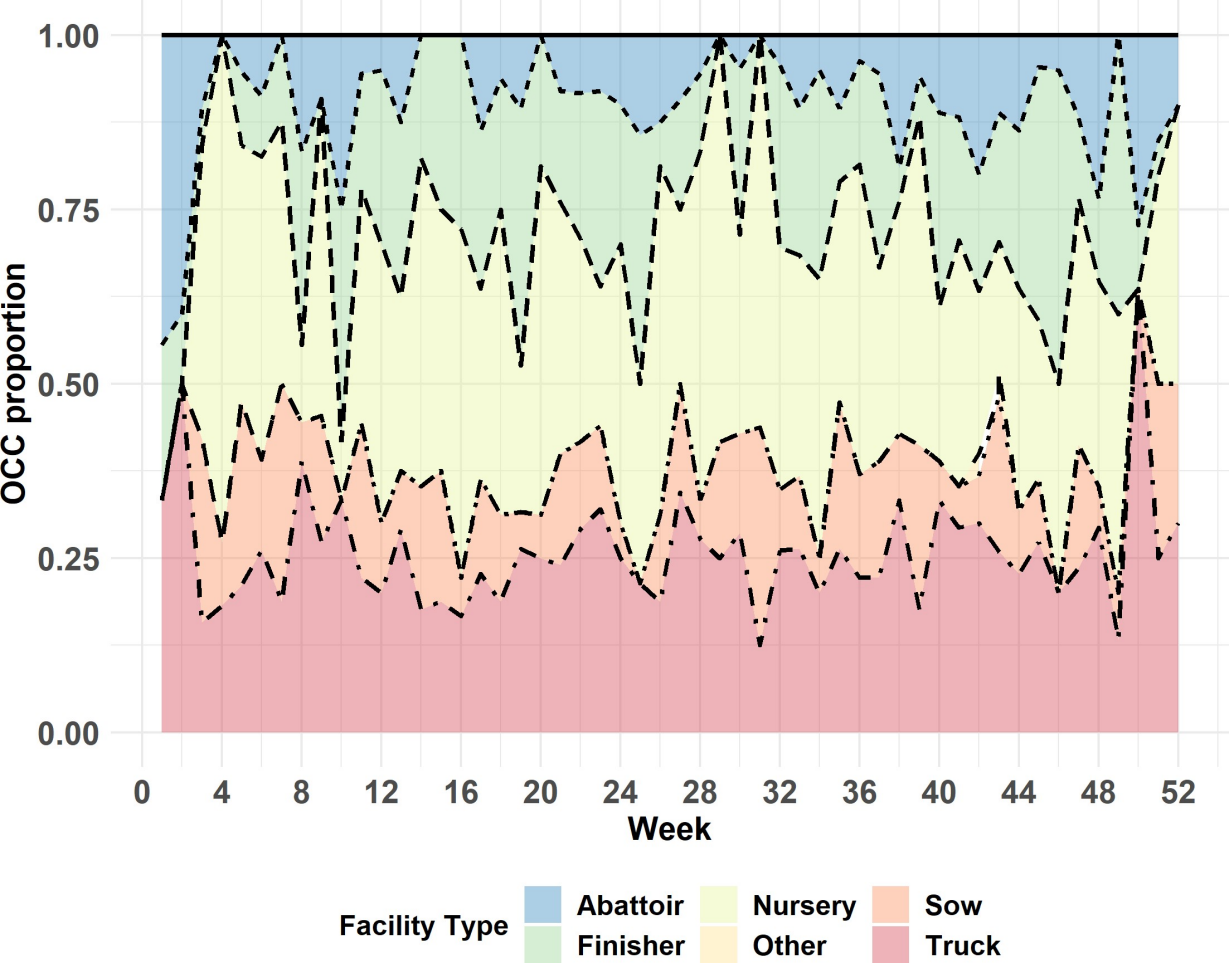
Proportional representation of each facility type within weekly maximum outgoing contact chain by week of the year, calculated on the basis of two-mode network.

**Fig 6:**
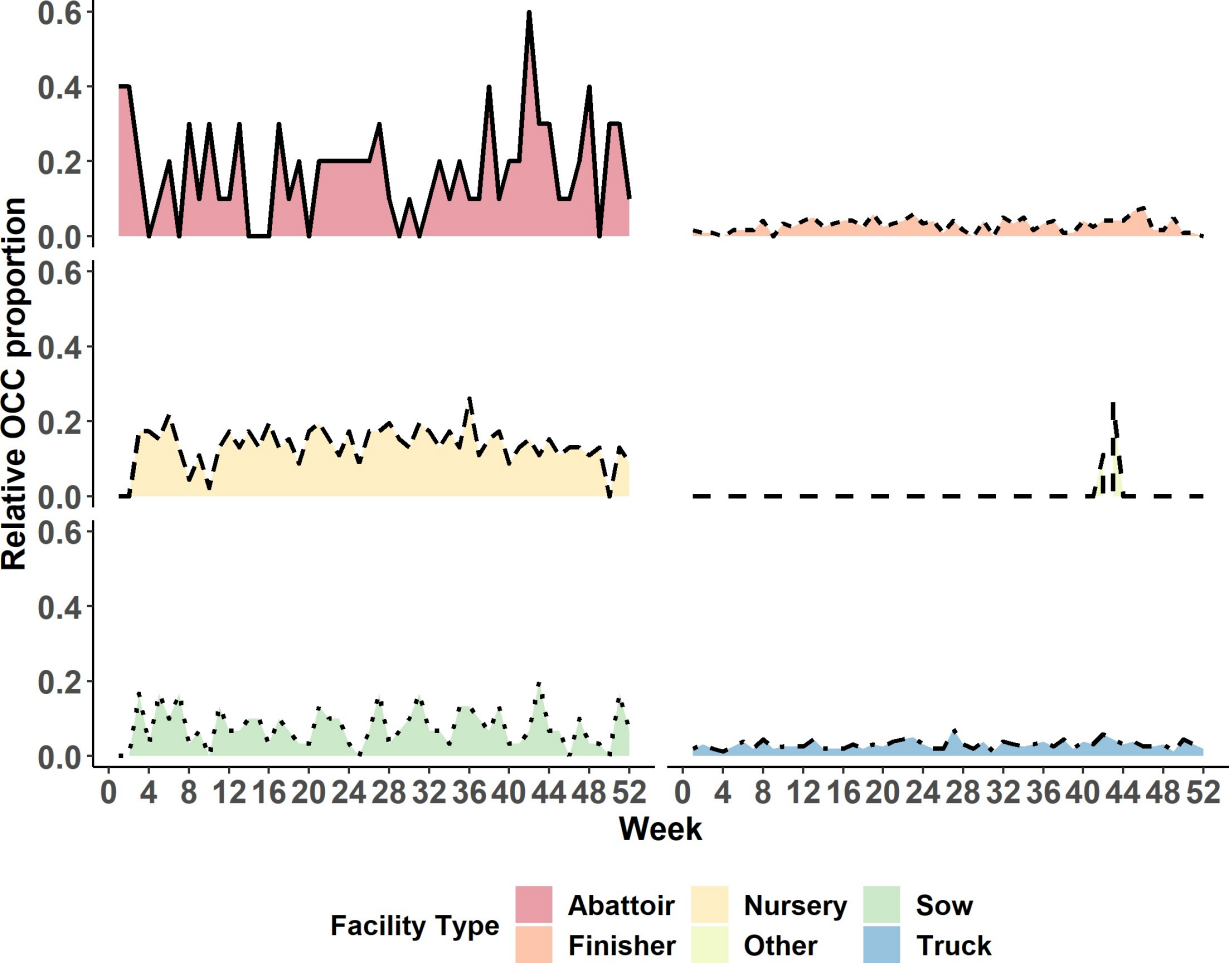
Relative occurrence of specific facility types in maximum weekly outgoing contact chain by week of the year, when expressed as a proportion of each facility type in the study population.

The weekly maximum OCC (MOCC_w_) size is plotted against week in Fig 4. There was a significant relationship with squared value of week across the 2015 year (p *=* 0.01), with the max chain length being 29 nodes. The proportion of each facility type in the MOCC_w_, when calculated using the number of facilities in each MOCC_w_ as a denominator has been provided in Fig 5. Similarly, the proportion of each facility type in the MOCC_w_, when calculated using the number of facilities of a specific type in the entire study population as a denominator, has been provided in Fig 6.

Predominantly trucks and nursery facilities were most frequent within the MOCC_w_s when only considering the populations within the MOCC_w_s (Fig 5). When relative proportions were considered (Fig 6), based on the total of each facility type within the network, nurseries were still present most frequently. However, abattoirs had peaks which surpassed the relative proportion of nurseries throughout the year (Fig 6).

### Daily one-mode networks: Components and demographics

**Fig 7:**
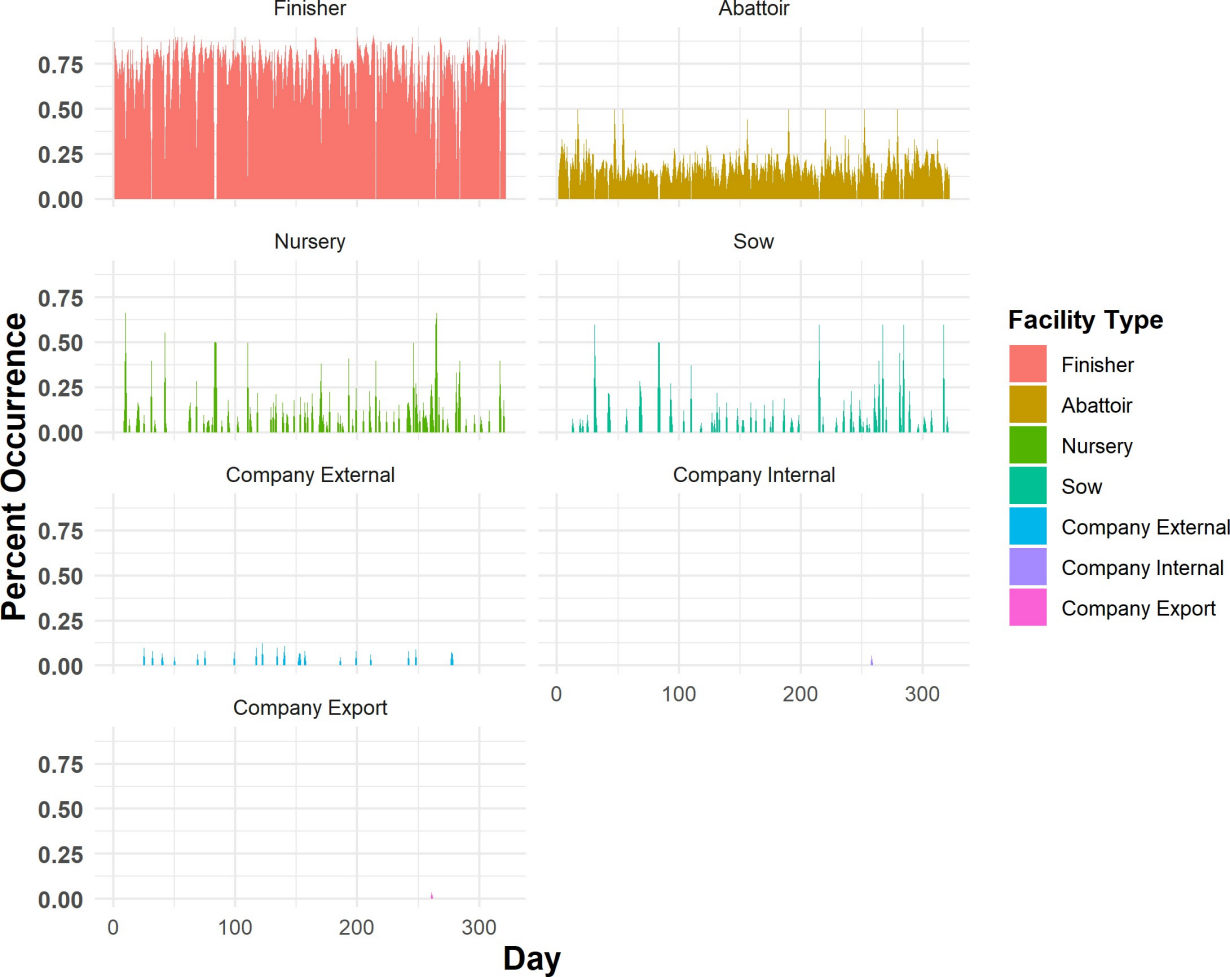
Daily one-mode network percent occurrence of facility types within the daily maximum weak component.

**Fig 8:**
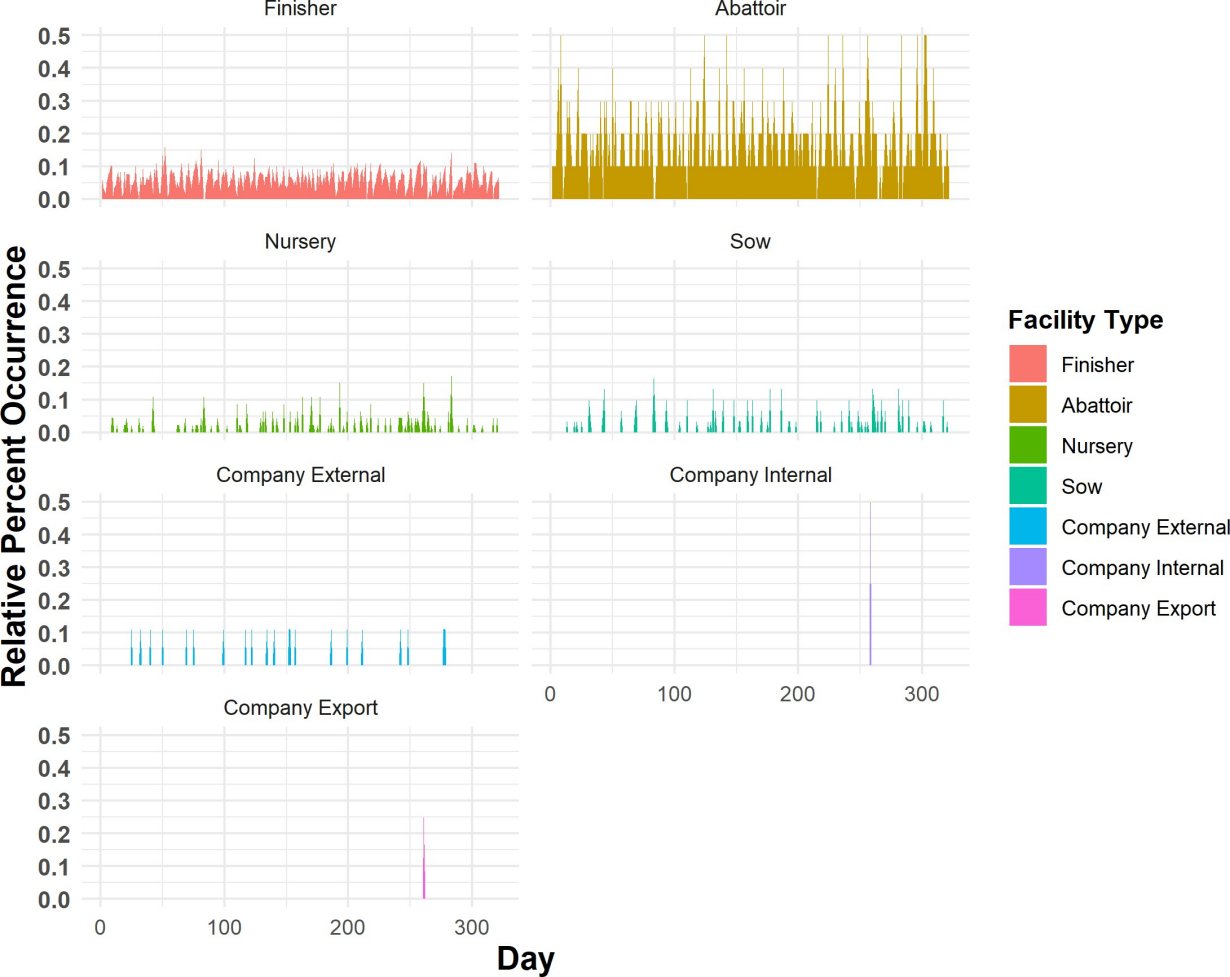
Daily one-mode network relative percent occurrence of facility types within the daily maximum weak component, calculated based on the total number of each facility type within the source population.

Daily one-mode network edges were based on undirected weak connections, therefore only weak components were investigated. The maximum WC_D_ ranged from 2 nodes to 30 nodes in size, the demographics of which can be seen in Figs 7 and 8. Fig 7 displays a percent occurrence of each facility type on a daily basis across the 2015 year. Fig 8 displays the same information, as a proportion of occurrence based on the total number of each facility type in the study population. The WC_D_ for one-mode networks did not display any significant linear or quadratic relationship with the day of the year.

## Discussion

Previous studies have noted that transportation vehicles contribute to the transmission of pathogens in swine populations [10,14,20]. Additionally, belonging to the same transportation system can increase the risk of disease transmission of specific genotypes of Porcine Reproductive and Respiratory Syndrome Virus (PRRSV) [21]. This finding along with transportation cleaning, washing and disinfecting that may be insufficient [22], emphasizes the need for understanding the role of transportation vehicles in swine movement and potential disease transmission.

Recent research utilizing network measures has shown applied methodologies on how understanding network connections can improve disease control programs [7]. Basic network measures such as degree status and “mean infection potential”, ingoing and outgoing contact chains, were utilized to estimate reductions in disease dissemination within the networks [7]. In this study we explored these measures, specifically outgoing contact chain among others, over different time periods. One, three, and seven days were selected based on the simplified assumption that proper sanitation of vehicles could be done over different time periods (i.e. one, three, or seven days). Furthermore, the addition of transportation vehicles as a node type within the network, has added a new degree of understanding to the impact of swine movements and potential disease transmission.

### Weekly two-mode networks: Weak component

The incorporation of trucks changed the magnitude of the weekly weak components within the same source population compared to a previous study which included only farms [4]. The weekly weak component was on average larger in size, which was expected since transportation vehicles have been directly incorporated in the analysis. However, the magnitude of the weekly weak component also increased significantly over time in size, for an estimated 0.4 nodes per week. Component sizes in general have been hypothesized to be an estimate of the size of outbreak populations [23–25]. Therefore, direct interpretation of this result is that there was a potential for the outbreak size to increase over time in this study population, when evaluated using this statistic. Similarly, the number of trucks with betweenness > 0 or with in/out-degree > 0 also increased linearly with time. These two results are in concordance because they suggest that the way that transportation vehicles were utilized in the study population was changing (i.e. the number of intensely used vehicles on a weekly basis is increasing), resulting in larger weak components. However, the number of trucks in the entire network has been increasing over time as well. If more vehicles were used equally across the network, we would expect a decrease in the size of the maximum weak component, via less shared connections between facilities and the trucks used for the shipments. The reason for such results could not be easily explained from the data available. It is possible that the utilization of transportation vehicles changed in the network as a response to one or more of the following issues: (i) disease outbreak, (ii) marketing conditions, (iii) change in biosecurity protocols (e.g, availability or affordability of truck washing stations), or (iv) increased use of vehicles on a part-time basis. In 2015, there were still some outbreaks of PED in Ontario (Canada) with herd-level prevalence at the end of 2015 being estimated at 2.25% [26]. Such conditions could influence the usage of transportation vehicles in the entire or in part of the network, which could result in the structural changes in the entire network.

### Weekly two-mode networks: OCC

Outgoing contact chains have been noted as being a good estimate for outbreak sizes [27]. Their advantage over weak components is that the direction of movements is incorporated into the analysis, and they embody a more representative estimate based on animal movements of the possible outbreak size in a given time period. Results from our regression analysis indicated that the weekly maximum outgoing contact chain demonstrated a quadratic relationship with week across the 2015 year. The longest weekly outgoing contact chains generally occurred during the summer months, suggesting that summer could be the time when disease can spread the furthest in this study population. This peak time of disease transmission and connectivity is supported by other regression results utilizing node level measures [4]. Current data did not allow for an investigation of possible reasons for such a finding, and we are not aware whether similar trends have been evaluated in other studies. However, this measure has been used as a means of mitigating disease spread via the theoretical removal of farms/nodes from the network [7]. In combination with the potential of seasonality in adherence to biosecurity practices, these results could indicate times of increased disease transmission and should be explored further.

### Two-mode network: Node centrality

As we expected, trucks, nurseries and finisher facilities had the highest betweenness values on average with trucks superseding the other facilities. The high betweenness value for nursery facilities was reported in other studies [28–31]. In this study, trucks were found to have an even higher betweenness than nurseries in this source population, supporting the concern that trucks represent an opportunity for potential disease transmission [12,27,32]. These results should, be considered together with the way that trailers and trucks themselves are being washed and disinfected. The fact that transportation vehicles are being in frequent contact with swine facilities is expected; the type of sanitation will primarily determine their potential for transmission of infection.

### One-mode daily network: Weak component

The daily weak components within the one-mode network show facilities that are related via the shared truck usage. Of the one mode network a maximum of 13.6% facilities (30/220), would be indirectly connected on a daily basis. Indirect routes of transmission via the transportation system have been reported in other studies, specifically, the classical swine fever outbreak in the Netherlands [33]. Knowing this information along with the knowledge that there are complications in terms of truck sanitation means that this is an area for further study in the swine industry [22,34].

### Limitations

The results reported here have some limitations, which are a consequence of the nature of the data and the way they were collected. The conversion of the two-mode network resulted in some facilities being lost due to a lack of information. Specifically, the lack of unique identifiers for the truck or truck company utilized by the farm facility itself.

Additionally, two important types of data that would be critical for a study like this are the infection status of individual farms with respect to important endemic diseases, and the actual date and quality of sanitation practice. Future efforts should be made to collect such information as it would greatly enhance our understanding how individual vehicles are used, and how the entire system functions.

Furthermore, the study network is incomplete. All the facilities within the data set are accounted for within the networks constructed. However, due to the movements to other populations by which the node could be a single facility or another production system entirely, the network could be theoretically much larger. We believe that incomplete networks could particularly influence node-level measures of abattoirs, because they receive animals from farms that were not part of this study population, node-level measures of transportation vehicles because they could be used to transport animals from other production systems, and network-level measures such as size of maximum weak components and outgoing contact chains.

Additionally, we assumed that trucks utilized at least once per year were part of the study population over the entire study period. However, it is also possible that at least some of these transportation vehicles could be used only intermittently. Consequently, the average in degree, out degree, and betweenness could be underestimated in our study. Despite such limitations, this study offers a unique perspective on the development of several network indices of interest for infection control in swine populations.

## Conclusion

The addition of trucks to a previously described network of farms resulted in the identification of a linear increase in the size of the maximum weekly weak component size over time. This finding may have been driven by the different usage of trucks over time. Despite known limitations of maximum weak components as an estimator of possible outbreaks, this finding suggests that transportation vehicles should be included, when possible and relevant, in the evaluation of contacts between farms.

The size of the MWC_w_ was between 3 and 123 nodes, which represented 0.8 to 32.8% of the total population in this network, and the demographic composition of MWC_w_ was relatively constant over time, with finisher sites being relatively the most frequent. The weekly maximum OCC (MOCC_w_) size ranged from 8 nodes to 29 nodes in a given week, corresponding to 2.1 to 7.7% of the networks’ population, respectively. With respect to node demographics, MOCC_w_ displayed larger between week variability than MWC_w_, with nursery facilities being relatively most frequent in majority of the weeks. The number of nursery and finisher sites with high betweenness values; and the size of the weekly MOCC_w_ all peaked in summer months, which warrants further investigations. Collectively, these statistics are suggestive of the maximum estimated extent of disease spread. Furthermore, they are indicative of the expected demographic proportions of affected sites over a single week in this, and in similar swine production systems.

## Supporting information

S1 Fig The total number of trucks that had contact with a specific combination of facility types in a single day. (TIFF)
