# Supplementary figures and images for: An investigation of transportation practices in an Ontario swine system using descriptive network analysis
Source: PLoS One. 2020 Jan 10;15(1):e0226813. doi: 10.1371/journal.pone.0226813 (PMC6953787; doi:10.1371/journal.pone.0226813)

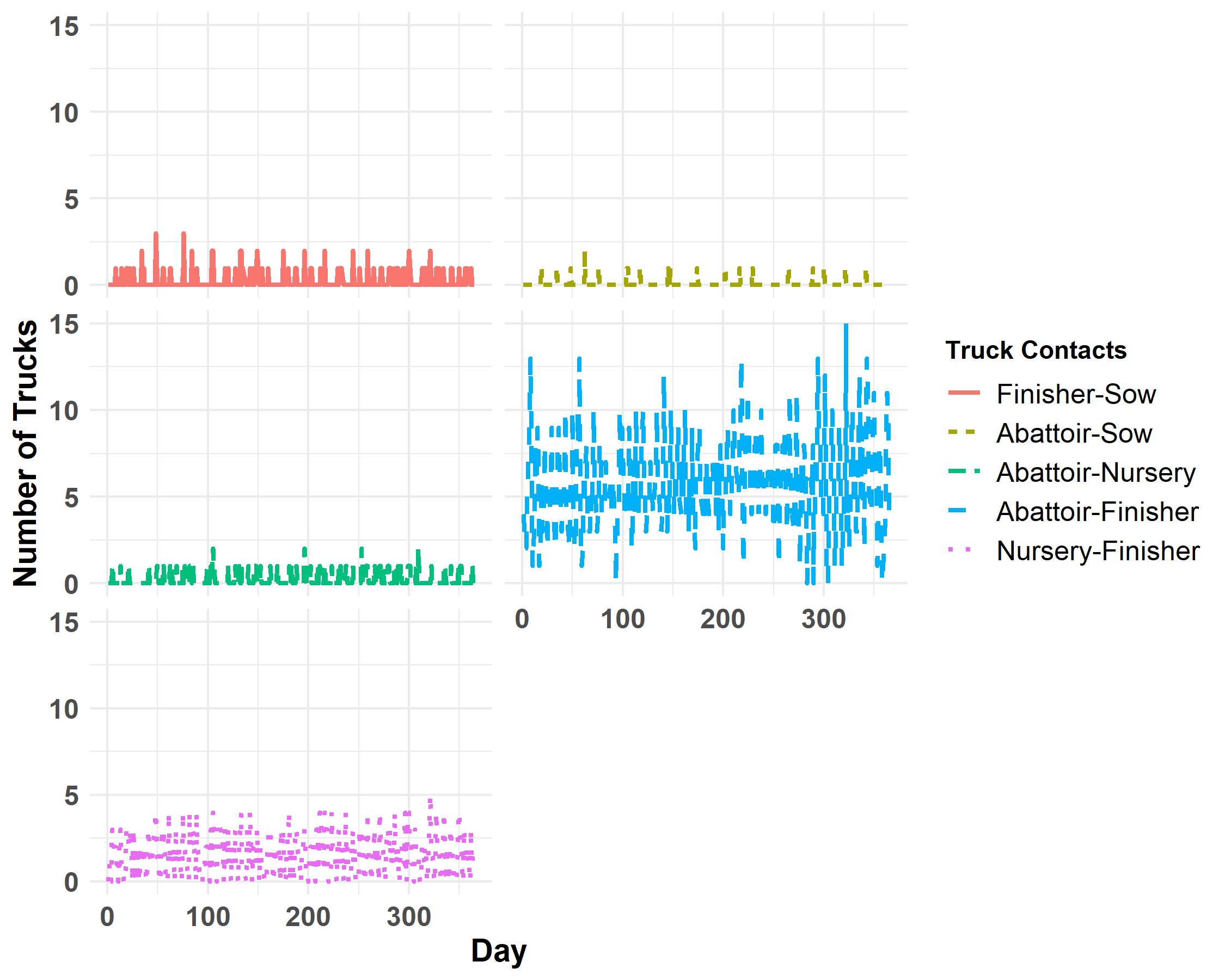

Supplement: S1 Fig — (TIFF) [file pone.0226813.s001.tiff]
